# Activation of hERG3 channel stimulates autophagy and promotes cellular senescence in melanoma

**DOI:** 10.18632/oncotarget.7831

**Published:** 2016-03-01

**Authors:** Mathew Perez-Neut, Lauren Haar, Vidhya Rao, Sreevidya Santha, Katherine Lansu, Basabi Rana, Walter K. Jones, Saverio Gentile

**Affiliations:** ^1^ Department of Molecular Pharmacology & Therapeutics, Loyola University, Chicago, IL-60153, USA

**Keywords:** potassium channels, autophagy, hERG, senescence, melanoma

## Abstract

Ion channels play a major factor in maintaining cellular homeostasis but very little is known about the role of these proteins in cancer biology. In this work we have discovered that, the Kv11.3 (hERG3) a plasma-membrane potassium channel plays a critical role in the regulation of autophagy in a cancer cell model. We have found that pharmacologic stimulation of the Kv11.3 channel with a small molecule activator, NS1643 induced autophagy via activation of an AMPK-dependent signaling pathway in melanoma cell line. In addition, we have found that NS1643 produced a strong inhibition of cell proliferation by activating a cellular senescence program. Furthermore, inhibition of autophagy via siRNA targeting AMPK or treatment with hydroxychloroquine an autophagy inhibitor activates apoptosis in NS1643-treated cells. Thus, we propose that, Kv11.3 is a novel mediator of autophagy, autophagy can be a survival mechanism contributing to cellular senescence, and that use of a combinatorial pharmacologic approach of Kv11.3 activator with inhibitors of autophagy represents a novel therapeutic approach against melanoma.

## INTRODUCTION

Autophagy is a ubiquitous lysosomal-dependent catabolic mechanism in which organelles (macro/micro-autophagy) or proteins (chaperon-mediated autophagy) are degraded [1]. Autophagy can function as a survival mechanism to support replenishment of primary biomolecules that are crucial for cellular growth but it can also activate a cell death pathway. Therefore, the role of autophagy in cell biology is still controversial and it appears to be specific to the cellular context or pathological condition [2]. Nutrient deprivation is the canonical stimulus for autophagy. The autophagic biochemical cascade begins with activation of the energy sensor AMP-activated kinase (AMPK) via phosphorylation of Threonine in position 172 (AMPK-pT172) [3] which in turn results in the inhibition of rapamycin (mTOR) activation and a direct phosphorylation of the Serine 555 on the ser/thr protein kinase ULK1 (ULK1-pS555) [4]. After induction, autophagy progresses by formation of membranous structures called autophagosomes that recruit the Light Chain 3 (LC3-I) protein which is subsequently cleaved at the carboxy terminus (LC3-II) [5]. LC3-II remains on mature autophagosomes until fusion with lysosomes is completed (autophagolysosome) and it is used to monitor progression of autophagy process [6]. Then, the content inside the autophagolysosome is degraded to simple biomolecules.

Changes in ionic gradient can regulate autophagy, suggesting that ion channels can play a major role during this event but data are surprisingly very limited and controversial. As variations of intracellular calcium plays a role in many cellular events, it was not entirely unexpected to find hepatocyte models in which autophagy can be inhibited by the removal of intracellular calcium [7]. Furthermore, the autophagic regulator AMPK, can be activated by the calcium-dependent kinase CAMKKII via direct phosphorylation suggesting that changes in intracellular calcium can activate autophagy [8]. In contrast, it has also been proposed that elevated intracellular calcium can activate mTOR resulting in inhibition of autophagy [9, 10]. Overall, these contradictory data suggest that cellular context dictate the role of calcium ions in autophagy [11] and that calcium channels alone are not sufficient to control this complex cellular event.

Moreover, increased activity of the mitochondrial ATP-sensitive potassium channel (mitoK_ATP_) has been associated with angiotensin-2 (Ang-II)-dependent autophagy in vascular smooth muscle cells [12]. These data suggest that changes in K^+^ gradients can play a role in autophagy. However, the role of mitoK_ATP_ or other K^+^ channels in autophagy remains to be characterized and to date, no surface membrane K^+^ channel has been linked with autophagy. Although incomplete, these seminal works suggest that fine regulation of ion channel activity is fundamental for the correct execution of autophagy.

Furthermore, in addition to nutrient deficiency, autophagy can be generated by several other stress factors in which ion channels play fundamental roles including, accumulation of damaged organelles/proteins and hypoxia [11, 13]. Cancer cells typically proliferate in an environment characterized by a shortage of nutrients and oxygen and recent studies demonstrate that cancer cells of different histogenesis use a variety of ion channels as important tools to respond to stress [14]. This suggests that ion channel activity and autophagy can be functional to the processes that cancer cells need to survive in a hostile milieu.

With their growing incidence worldwide, further understanding of the mechanisms underlying skin cancer and new possible targets for therapy is essential to improve the survival of advanced stage melanoma patients. Melanoma is the deadliest skin cancer in which multiple genetic alterations have been reported. However, dysregulation of the B-RAF gene via amplification or hyper-activation of the ser/thr protein kinase B-Raf protein (B-Raf^V600E^) in the Ras/Raf/Erk signaling pathways has been associated with acquisition of highly aggressive metastatic nature and poor prognosis in 80% of melanoma [14]. Current chemotherapies based on inhibition of B-Raf and its downstream effectors have offered limited success as high percent of tumors develop resistance [15]. Therefore, there is an urgent need to identify novel targets and therapeutic strategies against B-RAF-dependent melanoma.

The role of autophagy in cancer biology, including melanoma, is highly debated. The presence of B-RAF oncogene in melanoma has been associated with activation of autophagy and drug resistance [16]. It was proposed that autophagy is an important recycling mechanism that provides nutrients to sustain proliferation in melanoma. Conversely, other studies reported that activation of autophagy in B-RAF-dependent melanomas is accompanied by an increase in senescence-associated markers which suggests that autophagy can be a tumor suppressor mechanism that promotes permanent arrest of melanoma cell proliferation [17]. Overall, these works indicate that autophagy can play dual roles in cancer biology according to the cellular context and stage of the cancer.

It has long been known that melanomas express a series of ion channels including voltage-gated potassium channels (VGKC) and that changes in K^+^ ion gradients play a fundamental role in normal and cancer cell proliferation [18–25] but very little is known about the mechanisms through which K^+^ channels control this event. We found that a B-RAF-dependent human-derived melanoma cell line expresses the surface membrane homo-tetramer voltage-gated potassium channel Kv11.3. This channel is encoded by the KCNH7 (alias hERG3; human ether-a-go-go related gene 3) gene and it is traditionally known to play an important role in excitable tissues such as the brain [26, 27]. In our previous work we found that pharmacological stimulation of a VGKC that is similar to Kv11.3 determines a strong inhibition of proliferation in breast cancer cells [28]. In this work, we provide evidence that use of a small molecule Kv11.3 potassium ion channel activator, NS1643 determines autophagy in a B-RAF-dependent melanoma cell line without any significant effects in non-transformed skin cells. In addition, we show that NS1643-dependent autophagy is important for activation of cellular senescence in melanoma cancer cells. Furthermore, inhibition of NS1643-dependent autophagy leads to cell death by activation of apoptosis. Our data suggests for the first time that the surface membrane Kv11.3 potassium channel plays a major role in autophagy and that autophagy in melanoma can serve as survival mechanism for the acquisition of a cellular senescence phenotype. Finally, we propose that a combinatorial pharmacologic approach with Kv11.3 channel activator and autophagy inhibitors could be considered as a possible treatment strategy against melanoma.

## RESULTS

### Stimulation of the Kv11.3 potassium channel activity inhibits proliferation of melanoma cells

**Figure 1:**
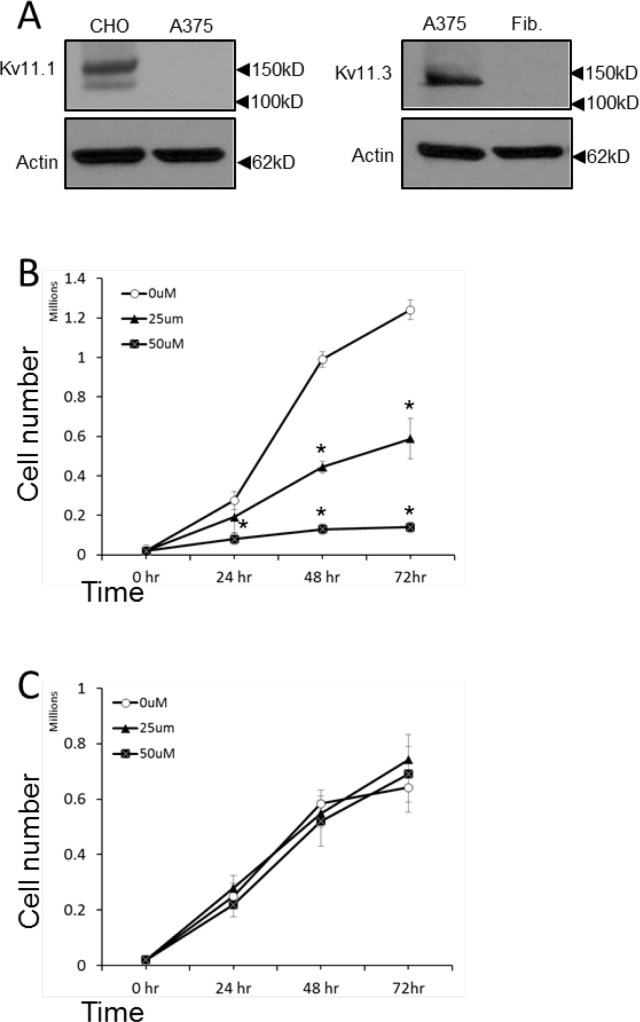
NS1643 inhibits melanoma cells proliferation **A.** Western blot showing expression of Kv11.1 in the overexpressing system CHO cells (control) and in A375 cells; Kv11.3 in A375 or normal forehead fibroblast (Fib.). **B.** A375 cells or **C.** normal fibroblast were treated with various concentrations of NS1643 or vehicle (DMSO; 0μM) for up to 72 hr. Data are expressed as mean ± s.e.m. from n=3. *p<0.01 (T-Test).

**Figure 2:**
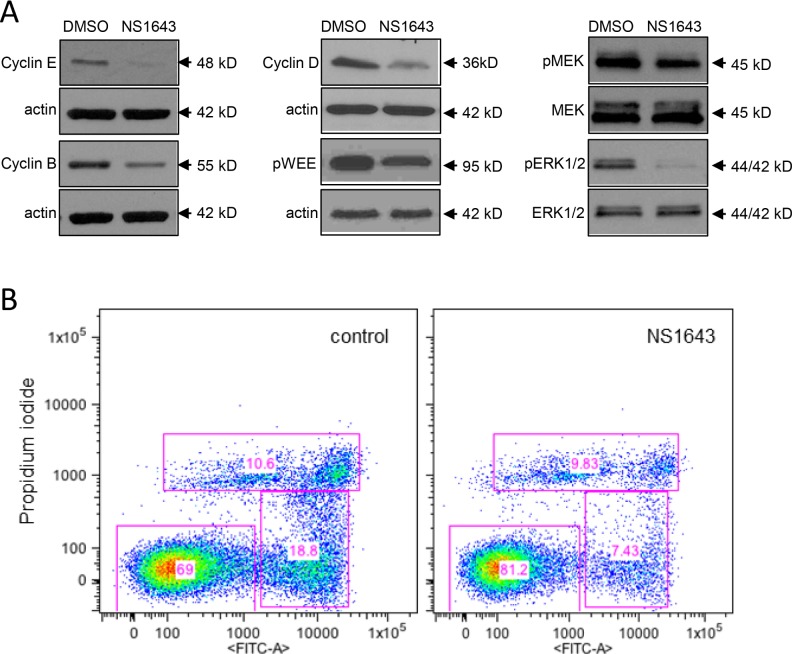
Stimulation of Kv11.3 downregulates proliferation pathways **A.** Representative Western blot analyses of extracts from A375 cells treated with DMSO (control) or NS1643 (50μM) for 48hr using antibodies against cyclin E, Cyclin B, Cyclin D, phosphorylated WEE1 and B) phosphorylated MEK, total MEK, phosphorylated ERK and total ERK. Data are expressed as mean SE from n=3. *p<0.01 vs untreated cells. **B.** Detection of apoptosis by AnnexinV apoptosis detection (Abcam; Cambridge, MA, USA) in A375 cells before ((Control) and after 48 h) treatment with 50 μM NS1643.

Melanocytes as well as neurons are derivative of neural crest cells. Consistently, melanoma cells can express voltage-gated ion channels (VGIC) which are known to play a major role in neurons as well as in cancer [14]. However, data on the identity and the role of these channels in melanoma is scarce. Previously, it has been shown that Kv11.1 channel, normally encoded by the human ether-a-go-go related gene 1 (hERG1) in heart [29], is critical in regulating cell proliferation of different cancer cell types and we found that stimulation of Kv11.1 with the pan-Kv11 activator NS1643 produced a significant inhibition of cancer cell proliferation. Interestingly, we did not detect expression of Kv11.1 in the human B-RAF-dependent melanoma cell line A375 (Figure 1A). Instead, we found that these cells expresses the neuronal Kv11.3 isoform [30]. Furthermore, the presence of Kv11.3 channel was not detectable in normal skin fibroblasts (Figure 1A). This suggests that, consistently with its neural origin, the melanoma cells retain the ability to express (or re-express) the neuronal isoform of the Kv11 family. To understand the effects of stimulation of Kv11.3 channel agonist NS1643 on proliferation of melanoma cells, we monitored proliferation rate of different groups of A375 cells treated with increasing amount of NS1643 for several time points (Figure 1B, 1C). Similar to the effect of siRNA targeting other members of the hERG channel family, suppression of Kv11.3 function produced non-viable cells [27, 31]. Therefore, we used normal skin fibroblasts as control for the effect of NS1643 on proliferation. We found that application of NS1643 determined a strong inhibition of A375 cell proliferation. In contrast, NS1643 did not exert any significant effects in skin fibroblast growth. These data suggests that stimulation of the Kv11.3 potassium channel can affect growth of B-RAF-dependent melanoma cells and that this effect is specifically mediated by Kv11.3 and not by unrelated targets. Notably, the number of A375 cells in plates that were treated with NS1643 remained constant after 24hr (Figure 1B) suggesting that stimulation of Kv11.3 determined arrest of the cell cycle specifically in A375 cells. To better characterize the effect of NS1643 on melanoma cell proliferation we monitored the expression level of proteins that are traditionally known to promote proliferation by regulating progression through the different phases of the cell cycle including cyclin E that is important for promoting G0/G1 to S phase transition, cyclin D that is important for progression through the S phase, cyclin B and Wee1 that are important for promoting S to G2/M transition. Western blot analyses revealed that application of NS1643 produced a strong down-regulation of all the detected cell cycle markers (Figure 2A) suggesting that the Kv11.3 opener inhibits proliferation by down-regulating expression of the cell cycle activators.

The Raf/MEK/ERK pathway is a major pathway involved in proliferation and melanomas are known to harbor activating mutations of B-RAF. Therefore, we monitored the activity of MEK and ERK in the B-RAF-dependent A375 cells before and after application of the Kv11.3 channel opener NS1643. Interestingly, we found that concomitant to the effect of NS1643 on cell cycle proteins, NS1643 strongly inhibited both MEK and ERK activities as indicated by the decreased phosphorylation of these proteins (Figure 2C). This suggests that, although there is mutational activation of B-Raf in A375 cells, activation of Kv11.3 channel results in reduced ERK activation and inhibition of cell proliferation.

### Kv11.3 agonist NS1643 activates cellular senescence in B-RAF-dependent A375 melanoma cells

**Figure 3:**
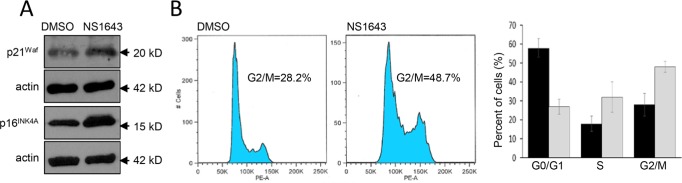
Stimulation of Kv11.3 activates a cellular senescence program **A.** Representative Western blot analyses of extracts (n=3) from A375 cells treated with DMSO (control) or NS1643 (50μM) for 48hr using antibodies against p21^Waf^ or p16^INK4A^. Actin immunoblots are utilized as loading controls. **B.** Flow cytometry analyses of DNA content of A375 cells treated for 48 h with DMSO (control) or NS1643 (50 μM). Histograms showing the percentages of cells at various phases of cell cycle in untreated cells (black bar; n=3) or treated with NS1643 (gray bars; n=3). Data are expressed as mean ± s.e.m. from n=3. *p<0.05 (T-Test).

In our previous works we have shown that stimulation of a Kv11.3 homologous protein, the Kv11.1 potassium channel, determined activation of a cellular senescence program in breast cancer cells [28]. In this work, we wanted to test the hypothesis that stimulation of Kv11.3 channel can induce senescence in a melanoma cell line with an oncogenic B-RAF. Therefore, we monitored senescent markers p21^Waf^ and p16^INK4A^ [32, 33] in A375 melanoma cells before and after application of NS1643 for different time points. We found that, in agreement with our previous investigation, stimulation of Kv11.3 strongly upregulated both senescent markers in melanoma cells (Figure 3A). However, in contrast with the effect of NS1643 in breast cancer cells in which we detected an arrest of the cell cycle in G0/G1 phase, analyses of the cycle progression in A375 melanoma shows that stimulation of Kv11.3 channel significantly increased the population of cells in the G2/M phase compared to untreated cells (Figure 3B) suggesting that NS1643 determined an arrest of the cell cycle in the G2/M phase of the cell cycle.

Overall, these data suggest that stimulation of Kv11.3 channel in B-RAF-dependent melanoma cell line activates a senescent program characterized by increased p21^Waf^ and p16^INK4A^ and arrest of the cell cycle in G2/M phase.

### Stimulation of Kv11.3 potassium channel stimulates autophagy via AMPK activation

**Figure 4:**
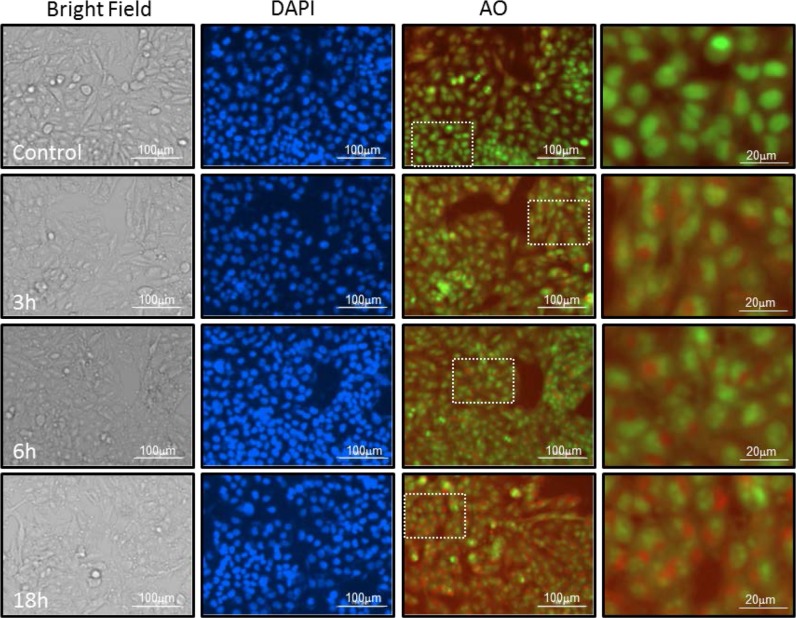
NS1643 increases formation of acidic vesicular organelles Representative Images of A375 cells treated with Vehicle alone or NS 1643 for 3, 6 and 18 hrs. Images of live cells under Bright filed, Nuclei stained with Hoescht-33342 (blue), acridine orange labeling of neutral pH cytosol and nucleus compartments (Green fluorescence) acridine orange labeling of acidic lysosomes (Red fluorescence). Dotted lines indicate areas that have been magnified. Cells are imaged live under 4x or 20x magnification on Cytation 3 Plate Reading Imager (Bioteck).

Studies on the crosstalk between senescence and autophagy are divisive and support both a positive relationship in which activation of autophagy leads to senescence and an inverse correlation in which inhibition of autophagy determines senescence [34, 35]. Autophagy is characterized by formation of Acidic Vesicular Organelles (AVO) that are detectable by vital staining with acridine orange (AO) [36]. We found that cells treated with NS1643 for 3,6,or 18 hours showed a progressive cytoplasmic accumulation of AVO (Figure 4), Indicating that stimulation of Kv11.3 channel could stimulate autophagy in B-RAF-dependent melanomas.

**Figure 5:**
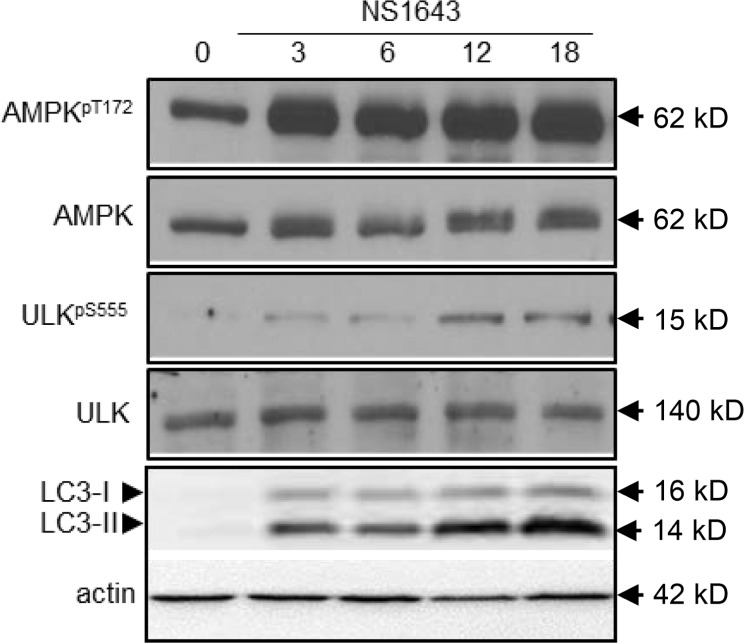
NS1643 activates autophagy **A.** Representative Western blot analyses of extracts (n=3) from A375 cells treated with DMSO (control) or NS1643 (50μM) for different time points as indicated using antibodies against phosphorylated (T172) AMPK, AMPK, phosphorylated ULK555, ULK, LC3.

To further investigate the effects of NS1643 on the regulation of autophagy we monitored activity of several autophagy markers such as AMPK, ULK1 and cleavage of LC3 before and after application of NS1643 for different time points via Western blot analyses. Interestingly, we found that NS1643 produced a quick activation of AMPK as indicated by an increase in phosphorylation at T172 (Figure 5) as early as 15 minute (Suppl. Figure 1) which was followed by an increased phosphorylation of ULK1 indicated by an increased phosphorylation of ULK1-pS555 (Figure 5). Furthermore, monitoring of LC3-I and LC3-II protein level before and after application of NS1643 revealed that stimulation of Kv11.3 channel resulted in a progressive increase in LC3-II levels that reached a plateau at 18hr. These data suggest that activation of the Kv11.3 potassium channel by NS1643 increases formation of autophagosomes in melanoma cells.

**Figure 6:**
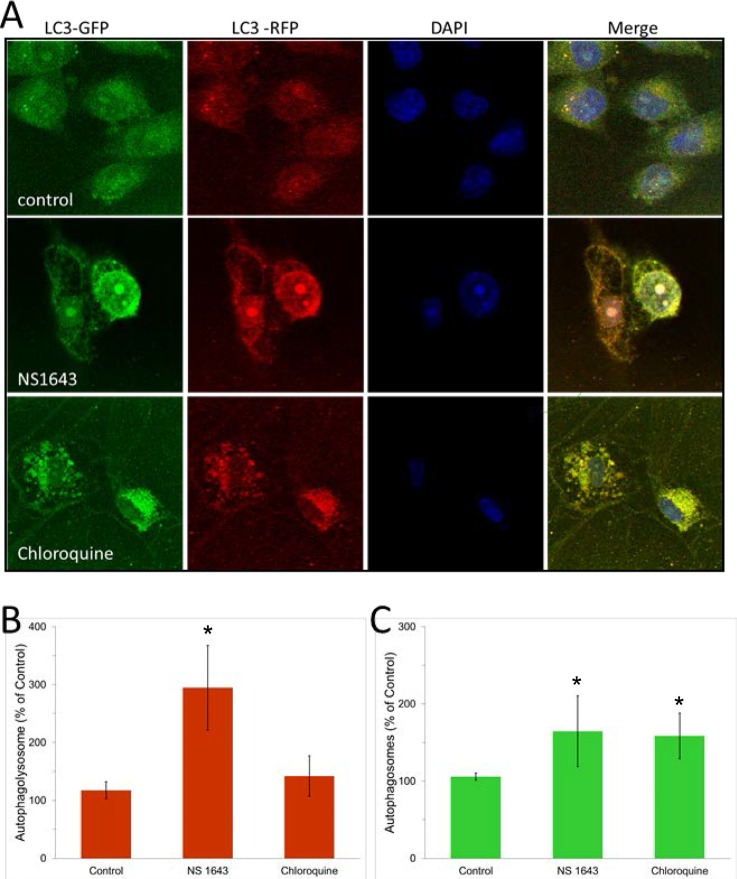
NS1643 stimulates autophagy flux Detection of autophagosome and autophagolysosme. **A.** Representative images of cells expressing RFP-GFP-LC3B construct followed by the treatment with vehicle alone or NS 1643 for 16 hours. Chloroquine treated cells served as a positive control. The nuclei are stained in blue. The natural pH LC3B positive autophagosome (green fluorescence) and acidic pH LC3B positive autophagolysosme (red fluorescence) were detected respectively using a confocal microscope. **B.** Quantification of LC3B positive autophagolysosome and **C.** Quantification (Image J, NIH) of LC3B positive autophagosomes in control, NS1643 or chloroquine treated cells. Data are expressed as percent change of control. Data are expressed as mean ± s.e.m. from n=3. *p<0.05 (T-Test).

The effect of NS1643 on autophagy markers could be related to protein accumulation due to impaired clearance of autophagosome or could be indicative of an augmented autophagolysosomes formation which suggests an increased autophagy flux. To discriminate between these, A375 cells were transduced with the autophagic flux detector Premo™ Autophagy Tandem Sensor and treated with NS1643 or with the autophagolysosome formation inhibitor, chloroquine. The Premo™ Autophagy Tandem Sensor construct encodes for an LC3II protein conjugated with Red Fluorescence Protein (RFP) in tandem with Green Fluorescence Protein (GFP). Based on the evidence that RFP is more stable in acidic condition compared to GFP, this system allows measurements of autophagolysosomal formation that indicates autophagic flux. Our experiments revealed that chloroquine treatment produced a significant increase of autophagosome but no changes in the formation of autophagolysosomes (Figure 6A, 6B). This confirmed the effect of chloroquine as autophagy inhibitor. In contrast, NS1643 treatment produced a significant increase of both autophagosome and autophagolysosome (Figure 6A, 6B, 6C) suggesting that stimulation of Kv11.3 channel results in increased autophagic flux.

### Autophagy is a survival mechanism for the NS1643-dependent senescence in melanoma cells

**Figure 7:**
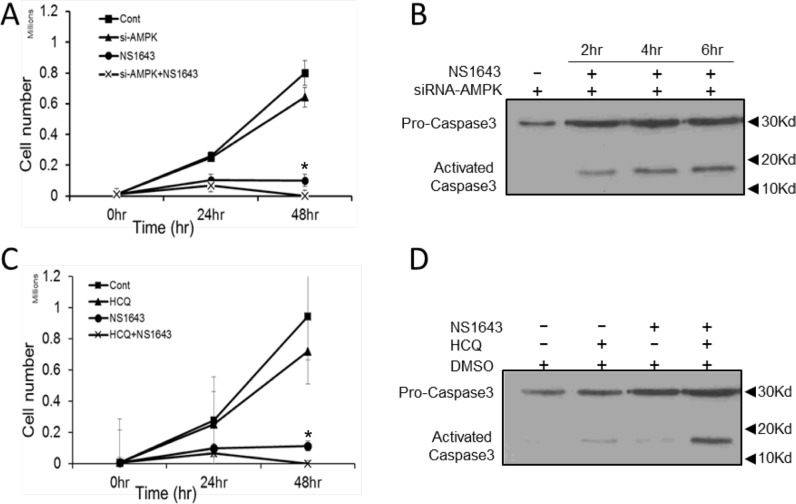
NS1643 activates apoptosis in the absence of autophagy **A.** Graph comparing the cell number over time of A375 cells treated with DMSO (Cont.; square), expressing siRNA targeting AMPK alone (si-AMPK; triangle), treated with NS1643 (circle) alone or expressing siRNA targeting AMPK and treated with NS1643 (cross). Data are expressed as mean ± s.e.m from *n* = 6 each treatment; *p<0.05 vs untreated cells; **p<0.05 vs siAMPK+NS1643 treated cells. **B.** Immunoblot analysis showing the effect of NS1643 on activation of caspase-3 at different times in cells in which AMPK function has been suppressed by si-RNA. **C.** Graph comparing the cell number over time of A375 cells treated with DMSO (Cont.; square), hydroxychloroquine alone (HCQ; triangle), NS1643 (circle) alone or HCQ+NS1643. Data are expressed as mean ± s.e.m from *n* = 6 each treatment; *p<0.05 vs untreated cells; **p<0.05 vs siAMPK+NS1643 treated cells. **D.** Immunoblot analysis showing the effect on activation of caspase-3 of NS1643 alone, HCQ alone or NS1643 + HCQ compared to untreated cells.

Our data suggest that stimulation of Kv11.3 channel in A375 melanoma cells initiate both cellular senescence and autophagy. To better understand the role of AMPK in the NS1643-dependent autophagy in melanoma cells, we monitored the effect of NS1643 in A375 cells in which AMPK protein activity was suppressed by siRNA-dependent gene silencing (A375-siAMPK). We found that although application of NS1643 in naïve A375 cells produced a strong inhibition of cell proliferation, surprisingly cell cycle inhibition was further potentiated when combined with AMPK knockdown (Figure 7A & Suppl. Figure 2). To further test whether this involved the cell death pathway we monitored activity of the apoptosis executioner caspase-3 before and after application of NS1643 in A375-siAMPK cells. We found that in A375-siAMPK cells, NS1643 induced apoptosis as indicated by increased cleavage of caspase-3 (Figure 7B). Importantly, these data suggests that, at least in cells treated with a potassium channel activator, there is a functional relationship between autophagy and cellular senescence in which autophagy serves as a survival mechanism.

**Figure 8:**
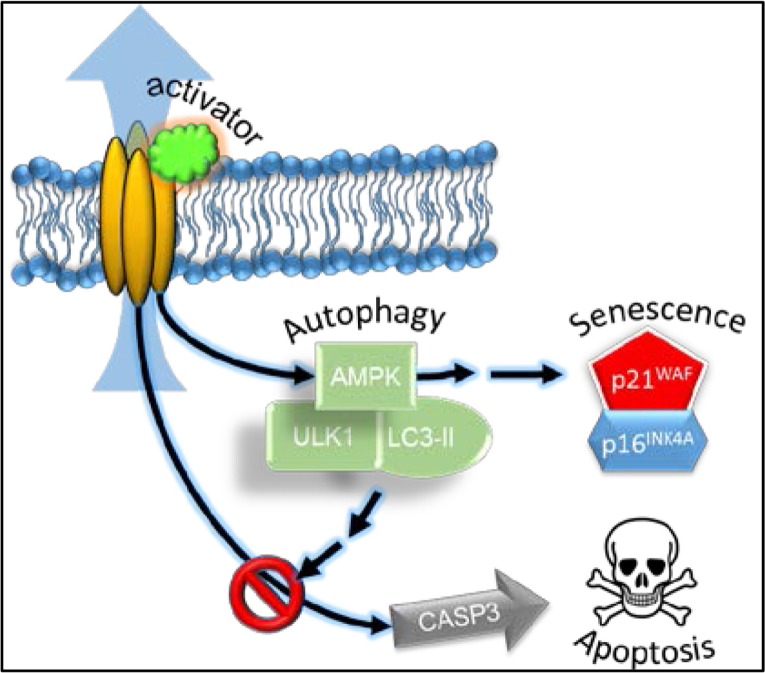
Schematic representation of the effect of the Kv11.3 potassium channel activator in A375 melanoma cell Stimulation of Kv11.3 channel leads to activation of AMPK via an unknown mechanism. NS1643-dependent AMPK-mediated autophagy is functional for cellular senescence as a survival mechanism possibly by inhibiting caspase-3-dependent apoptosis and cell death.

Common therapies for B-RAF-dependent melanoma, which include radiation, surgery and chemotherapy, become less effective and progressively more toxic [15]. There is a need for new pharmacologic approaches and novel targets that improve the standard of care. Therefore, based on our data we hypothesized that pharmacologic inhibition of NS1643-dependent autophagy could promote cell death in melanoma cells. We tested this hypothesis by using a combinatorial drug approach in which we treated A375 cells with NS1643 and the FDA approved antimalarial and autophagy inhibitor Hydroxychloroquine (HCQ; Plaquenil ®). Interestingly, we found that application of HCQ alone did not exert any significant effects on proliferation rate of A375 cells (Figure 7C). However, when HCQ was administered in combination NS1643, a progressive reduction in cell number and an increased caspase-3 activation was observed (Figure 7C, 7D) suggesting that the combination of NS1643 and HCQ determined activation of the apoptotic pathway.

## DISCUSSION

It has been known for decades that the change in ionic gradients confers the ability of a cell to activate, arrest and modulate complex biochemical pathways that are fundamental for virtually all cellular events. This indicates that ion channels and their synergistic activities are among the most important factors in establishing and maintaining cellular homeostasis in normal and pathologic conditions. Several studies have revealed that cancer cells of different histogenesis can express a variety of ion channels, including members of the human ether-a-go-go related gene (alias KCNH) family [23]. However, the role of these proteins in cancer biology is severely understudied.

Research in which a member of the hERG family, the Kv11.1 potassium channel, have been pharmacologically inhibited demonstrated that this protein plays a critical role in cancer biology [37]. However, hERG channel blockers are recognized as high risk medication because of their important side effects especially in the heart and the acceptance of these drugs is very limited [38] [29]. In contrast, stimulation of hERG channels appears to be well tolerated [39, 40] and the effects of hERG openers on cancer cells are emerging as a new target for treatment of hERG expressing cells [21, 27, 28]. In this work, our experiments confirmed our previous findings [28] that the pan-Kv11 potassium channel activator NS1643 that targets the Kv11.3 channel expressed in melanoma cells, significantly inhibited proliferation without activating cell death, reduced expression of cyclins and significantly up-regulated the tumor suppressors p21^Waf^ and p16^INK4A^. Both, p21^Waf^ and p16^INK4A^ are also considered markers of cellular senescence, which is characterized by a permanent arrest of the cell cycle, suggesting that the stimulation of potassium channels can activate a potent tumor suppressor mechanism in melanoma cells. In contrast with the effect of NS1643 in the breast cancer cell cycle, stimulation of Kv11.3 in melanoma cells by NS1643 produced a cell cycle arrest in G2/M phase. This result was unexpected as it is generally accepted that senescent cells arrest in the G0/G1 phase of the cell cycle [41]. However, studies from other groups have reported that senescence-inducing stimuli can activate a p21^Waf^-dependent signaling in G2 phase resulting in slippage of long-term G2 phase (6 days) into G1 phase without mitosis. Therefore, we can speculate that NS1643-treated cells could slip into the G0/G1 phase without dividing. To test this hypothesis more specific experiments will be performed that are at this time not the focus of this work.

Interestingly, in the process of our experimentation, we observed that cells treated with NS1643 revealed the presence of intracellular membranous formations that suggested activation of autophagy. Autophagy is a catabolic degradation event that is essential to cellular homoeostasis but surprisingly, very little is known about the contribution of ion channels to autophagy. As in some circumstances activation of cellular senescence is associated with autophagy, we tested the possibility that stimulation of Kv11.3 channel would lead to upregulation of autophagy markers. In our initial study we found that treatment with NS1643 determined a strong increase of the senescence and autophagy markers. These results are important because they show for the first time, that changes in cytosolic K^+^ gradients via a surface –membrane K+ channel contributes to two major cellular events such as autophagy and activation of a senescence-like program. Further investigations revealed that the NS1643-dependent increase in autophagy markers preceded the NS1643-dependent activation of a cellular senescence-like pathway suggesting that in cells which undergoes potassium loss, autophagy can function as precursor event for permanent arrest of cell proliferation.

It has been established that when cells are in a nourishing environment, the nutrient sensor AMPK is inactive and autophagy is kept at basal level. In contrast, during the process of nutrients depletion, production of AMP increases. Binding of AMP to AMPK determines a conformational change of the protein that exposes T172 to the activity of ser/thr kinases resulting in activation of AMPK. In normal culturing conditions, cells can experience nutrient depletion after several days if medium is not replaced. Interestingly, we found that application of NS1643 to cells that grow in complete medium induced a rapid phosphorylation of AMPK (30min) on T172 suggesting that stimulation of Kv11.3 channel activates autophagy via a mechanism that is independent of nutrient deficiency.

Several studies have reported that increased intracellular calcium can promote autophagy and that T172 can be a target of the Ca^2+^/calmodulin-dependent ser/thr kinase CAMKKII. Interestingly, similar to the effect of NS1643 in breast cancer cells, stimulation of Kv11.3 produced a rapid increase of intracellular calcium (Suppl. Figure 3A). This phenomenon can be explained by the fact that stimulation of K^+^ channels (which results in loss of intracellular potassium) causes hyperpolarization of the cells (increased negative charge) that, in non-excitable cells such as melanoma, provides a driving force for calcium entry [27]. Therefore, we hypothesized that NS1643-dependent increase of cytosolic calcium gradient determines a CAMKKII-dependent AMPK activation.

However, experiments in which NS1643 was added to cells growing in a Ca^2+^-free medium or in the presence of the pan-Ca^2+^-channel blocker cobalt, did not reveal any significant inhibition of AMPK phosphorylation on T172 suggesting that NS1643-dependent autophagy or AMPK activation is independent of changes in intracellular calcium (Suppl. Figure 3B). Therefore, at this time, the identity of a ser/thr kinase or phosphatase that can directly activate AMPK upon changes in ionic gradients remains unknown and a more extended study needs to be implemented. Remarkably, a recent study showed that depolarization (which occurs when the membrane collects more positive charges in the intracellular space) produced changes in phospholipid phosphorylation [42]. In addition, a previous study reported the expression of a Voltage Sensitive (lipid) Phosphatase [43] (VSP; activated by depolarization and inactivated by hyperpolarization) on the surface membrane of several species including humans. Although these important discoveries need to be further developed, it is well accepted that changes in lipid phosphorylation can strongly influence intracellular signaling for example, by promoting activity of protein kinases containing a pleckstrin homology (PH) domain or protein phosphatases in complex with PH-containing proteins [44]. Therefore, we speculate that a possible mechanism linking Kv11 channel's activation to AMPK could be possible via t NS1643-dependent hyperpolarization mediated phospholipid phosphorylation by inhibiting “a” VSP-type protein activity. This would result in either stimulation or inhibition respectively of kinases or phosphatases that target AMPK.

Interestingly, suppression of AMPK function produced NS1643-dependent activation of caspase-3 and apoptosis. This suggests that AMPK appears to be critical in maintaining cell viability in the NS1643-treated cells by promoting autophagy as a survival mechanism. We don't know at this time what is the mechanism through which NS1643-dependent AMPK activation controls caspase-3. However, it has been suggested that AMPK can stimulate NF-kB-dependent protein synthesis of BCL2 and Survivin [45] which are two potent inhibitors of caspase-3 suggesting that stimulation of Kv channels activity might activate NFkB-dependent survival mechanism.

Finally, Kv channels have also been found capable of activating intracellular signaling independently of their ability to conduct K^+^. For example, mutations that eliminated the ion flux of the Kv10.1 channel (a member of the superfamily of ether-a-go-go channels) did not alter its ability to activate p38MAPK (another autophagy regulator) [46–48]. In contrast, mutations that forced the channel in an open state inhibited p38MAPK activity suggesting that Kv channels can function as voltage sensors to indirectly control activity of key players of autophagy as well as other biochemical pathways.

The role of autophagy in cancer is still highly debated. A group of studies support the hypothesis that autophagy is a tumor suppressor mechanism as the removal of damaged and/or obsolete proteins during autophagy can reduce genomic instability, a potent tumorigenic trigger. In addition, autophagy defects are associated with increased risk of cancerogenesis [49].

In contrast, other studies demonstrate that the critical role of autophagy in degrading and recycling cellular components in response to stress can favor cancer cell survival in a nutrient deficient and/or hypoxic environment in which typically cancer cells proliferate [50]. In addition, it has been shown that oncogenic B-RAF induces autophagy to confer drug resistance in melanoma [51]. Therefore, autophagy can confer stress tolerance to cancer cells and promote tumorigenesis.

Results from our experiments provide evidence for a third scenario in which autophagy is both a tumor suppressor because it is required for the correct execution of cellular senescence and confers survivability properties because it inhibits cell death. However, at this time we don't know whether autophagy is necessary for the activation of senescence and more investigations in this direction need to be developed.

Although overcoming senescence could be considered an important step that can potentially be associated with malignancy, there is no evidence that cells can naturally escape senescence other than with artificial suppression of senescent markers. Therefore, although senescence and autophagy appear to be regulated by distinct biochemical pathways, they share common characteristics (e.g. promote survivability) which suggest that these events can function synergistically to maintain homeostasis.

Overall, our data suggest that stimulation of a surface membrane potassium channel confers to cancer cells a non-replicative immortality by using autophagy (Figure 8). This phenomenon could offer a clinical advantage based on the facts that cellular senescence is a permanent arrest of the cell cycle and that blocking autophagy in senescent cells leads to cell death.

Furthermore, studying the effects of Kv11 openers on cancer cells can lead to identification of novel therapeutic targets such as autophagy markers and based on our results, we propose that a combinatorial approach with Kv11 channel activators and autophagy inhibitors could be considered as a potential cytotoxic strategy against Kv11 positive cancers.

## MATERIALS AND METHODS

### Cell culture

Human melanoma cell line, A375 (ATCC) was maintained at 37°C and 5% CO_2_ in Dulbecco's modified Eagle's medium (DMEM 4.5 g/L glucose) supplemented with 10% fetal bovine serum, non-essential amino acids, Penicillin (100 μg/ml) and streptomycin (100 μg/mL) antibiotics (complete media).

### Antibodies and reagents

Rabbit Anti AMPK, p-AMPK ^T172^, Pro-Caspase 3, ULK, p-ULK ^S555^, p21^WAF^, cyclin E, D, & B, MEK, p-MEK, ERK 1/2, p-ERK ½ and pWEE antibodies were purchased from Cell Signaling Technologies, Inc. (Boston, MA, USA). p16INK4A was purchased from Abgent (San Diego, USA, CA). The Kv 11 agonist NS1643 was purchased form Alomone Labs (Jerusalem, Israel).

### Cell proliferation assay

A375 cells were plated in 24 well plates and cultured in DMEM supplemented 10% FBS, NEA and antibiotics. Sub confluent cultures of A375 cells were treated with NS1643 at 50 μM concentration. Following treatment up to 72 hours, at respective time points the cells washed with 1X PBS, trypsinized with 0.25% trypsin-EDAT (Life Technologies) and collected in 1.5 mL centrifuge tubes. The cells were collected by centrifugation at 3000 rpm for 5 min and suspended in complete medium. Cell suspension was incubated with 0.4% Trypan blue in 1.1 ratio for 3 min and 10ul of cell suspension was counted using a hemocytometer and cell counter (Countess; Invitrogen).

### Flow cytometry

A375 cells, control and treated with NS 1643 were subjected to cell cycle analysis by flow cytometry. Following treatment, the cells were washed with 1X PBS, trypsinzed with 0.25% Trypsin-EDTA, centrifuged at 3000 rpm for 5 min. The cell pellet was washed once with 1X PBS and re-suspended in ice cold 70% ethanol. The cells were stored at −20°C until samples from all time points were collected. One million cells from each sample were processed for flow cytometry. Cells were washed and re-suspended in 1X PBS, incubated with RNase A (Promega, Madison, WI, USA) at 0.5 μg/mL concentration and Propidium iodide (Sigma – Aldrich; 50μg/mL) for 2h at 4°C. Propidium iodide labeled cells were detected with flow cytometry and the cell cycle analyzed using FlowJo software (Tree Star Inc.; Ashland, OR, USA).

### Western blot analysis

Control and NS 1643 treated A375 cells were harvested by trypsinization with 0.25% Trypsin-EDTA, washed in PBS, and lysed with cold radioimmuniprecipitation assay (RIPA) buffer [50 mM Tris HCl (pH 8.0), 150 mM NaCl, 1% Tergitol (Sigma-Aldrich, St. Louis, MO, USA), 0.5% Na-deoxycholate, 0.1% SDS, 1 mM phenylmethylsulfonyl fluoride, 1 mM NaF, 1 mM Na3VO4, and 1 × Protease Inhibitor Cocktail (Sigma-Aldrich)]. Protein concentration was determined by BCA assay (Thermo Scientific). Equal amount of protein samples were incubated with 4X Laemmli buffer at 95°C for 5 min. The samples (32 μg) were subjected to SDS-polyacrylamide gel electrophoresis on precast 4–15% gradient mini-gels (Bio-Rad, Hercules, CA, USA) and transferred onto a nitrocellulose membrane. Membranes were blocked with 5% nonfat milk in Tris-Buffered Saline (Sigma-Aldrich) containing 0.1% Tween 20 (Sigma-Aldrich) (TBST). Following washes with TBST the membranes were incubated overnight at 4°C with primary antibodies diluted 1:1000 (p16INK4a dilution was 1:500) in 5% bovine serum albumin in TBST. The membranes were then washed with TBST and incubated with anti –rabbit secondary antibody (1:2000) in TBST with 5% nonfat milk for 1 h at room temperature. Following secondary incubation the membranes were thoroughly washed with TBST and visualized using Super Signal West Pico Chemiluminescent Substrate (Thermo Scientific, Pittsburgh, PA, USA). LC3 blots were done on 12% in house gels using RIPA isolation, 5% milk blocking and 1:1000 primary AB (Cell Signaling 2775) 1:3000 secondary Cell Signaling).

### Supravital cell staining with acridine orange

for autophagy detection/Detection of acidic vesicular organelles (AVOs). A375 cells were plated in 12-well plates and cultured in complete DMEM. Following treatment with NS 1643 (50μM) for the indicated time points the cells were stained with acridine orange (Life Technologies) at a final concentration of 1μg/mL for 15 min. Acridine orange labels cytoplasm and nucleus with fluorescent bright green and the acidic compartments with fluorescent red, with the intensity of red being directly proportional to acidification of the cellular compartments. Hoescht-33342 (Life Technologies) stain was performed with 5ug/mL media. Following staining, media was removed, cells were washed with complete medium and fluorescent images were captured for green nuclear and cytosol labeling (525 nm), red acidic vesicular labeling (647 nm) and blue for nuclear Hoescht 33342 (447 nm) excitation wavelengths respectively. Imaging was performed on Cytation 3 Imaging Reader with 20x and 4x objective magnification as indicated in the figure.

### Detection of autophagic flux

The formation of autophagosome and autophagolysome in A375 cells in control and NS 1643 treated cells was detected using the Premo™ Autophagy Tandem Sensor RFP-GFP-LC3B Kit (Thermo Fischer Scientific, Grand Island, NY, USA) as described in the manufacturer's instructions. The RFP-GFP-LC3B sensor enables the detection of LC3B positive, neutral pH autophagosomes in green fluorescence (GFP) and LC3B positive acidic pH autophagolysosme in red fluorescence (RFP). The cells were grown on coverslips and incubated with 6 μL of BacMam Reagents containing the RFP-GFP-LC3B overnight. The cells were then treated with either vehicle alone, NS 1643 at 50 μM concentration, or hydroxychloroquine sulfate (CalBiochem) for 16 hours. The cells were rinsed in 1X DPBS, nuclei were stained with DAPI The coverslips were mounted with VECTASHIELD reagent (Vector Laboratories, Burlingame, CA) and fluorescent images were taken using confocal microscopy (Carl Zeiss Meditec, Inc., Thornwood, NY). LC3B positive autophagosomes (green) and LC3B positive autophagolysosome (red) were analyzed and quantified using Image J.

## SUPPLEMENTARY FIGURES


