# Supplementary material for: Activation of hERG3 channel stimulates autophagy and promotes cellular senescence in melanoma
Source: Oncotarget. 2016 Mar 1;7(16):21991–2004. doi: 10.18632/oncotarget.7831 (PMC5008339; doi:10.18632/oncotarget.7831)
Supplement: Supplementary file 1 [file oncotarget-07-21991-s001.pdf]

SUPPLEMENTARY FIGURES

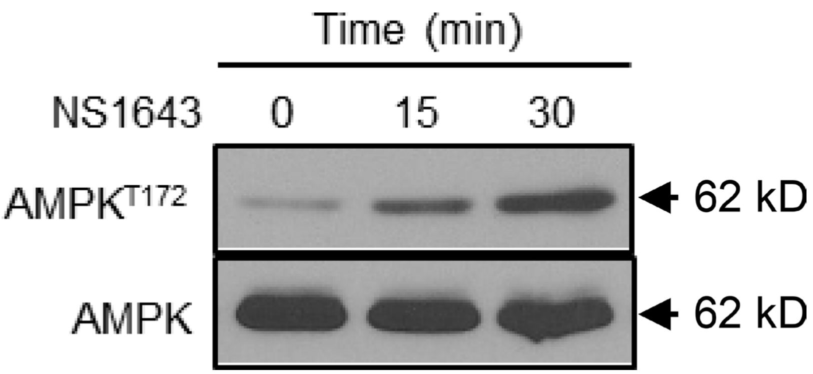

Supplementary Figure S1: Western blot showing NS1643-dependent early activation of AMPK.

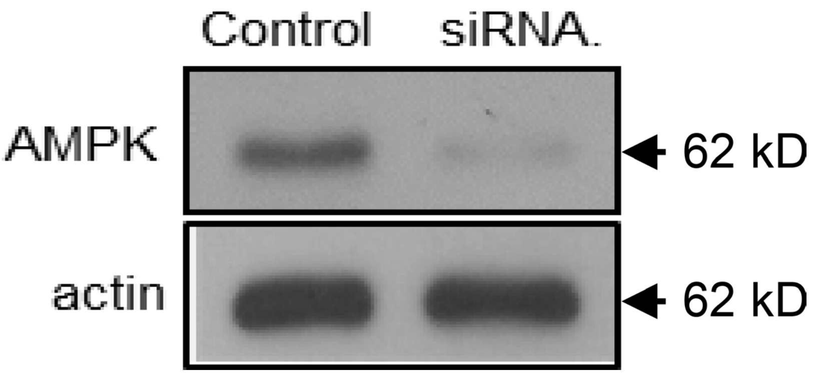

Supplementary Figure S2: Western blot showing the efficacy of siRNA targeting AMPK in A375 melanoma cells.

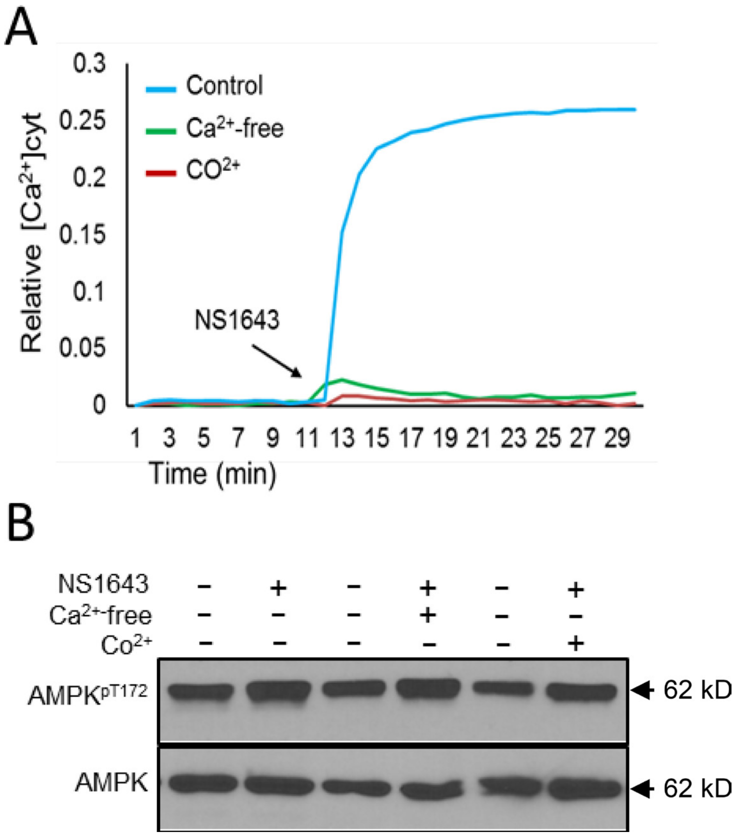

Supplementary Figure S3: A. NS1643-induced increases in cytosolic [Ca^{2+}], as measured by the Fura-2 ration (F340/F380) in A375 cells bathed in regular ringer (blue trace), Ca^{2+}-free ringer (green trace) or regular ringer with 10µM Co^{2+} (red trace). [Ca^{2+}] measurements with fura-2 was performed as previously described in [52]. Baseline luminescence was recorded every minute for 30 min using a PHERAstar plate reader (BMG Labs). **B.** Western blot showing the effect of NS1643 on AMPK activation in cells exposed to regular ringer, Ca^{2+}-free ringer, or regular ringer with 10µM Co^{2+}.
